# Venoplasty and Venous Stenting in Patients with Chronic Venous Insufficiency in the Lower Extremities

**Published:** 2016-10-03

**Authors:** Majid Moeini, Mohammad Reza Zafarghandi, Morteza Shahbandari, Azadeh Sayarifard, Morteza Taghavi, Javad Salimi, Pezhman Farshidmehr, Mohammad Hasani, Mohammad Reza Tobaei

**Affiliations:** 1 *Sina Hospital, Tehran University of Medical Sciences, Tehran, Iran. *; 2 *AL-Zahra Hospital, Isfahan University of Medical Sciences, Isfahan, Iran.*; 3 *Center for Academic and Health Policy, Tehran University of Medical Sciences, Tehran, Iran.*

**Keywords:** *Venous insufficiency*, *Lower extremity*, *Endovascular procedure*

## Abstract

**Background: **Venoplasty and stenting is a minimally invasive therapy that can be used for patients with deep venous insufficiency in the lower extremities. This study aimed at investigating the effect of venoplasty and venous stenting in patients with chronic venous insufficiency in the lower limbs.

**Methods**: This prospective case-series study recruited patients with chronic deep venous insufficiency in the lower limbs candidated for venoplasty in the Vascular Clinic of Sina Hospital in Tehran, Iran. Venoplasty and stenting was done if the deep venous system in the lower extremities had stenosis or obstruction on venography. The patients were visited 1, 3, and 6 months after venoplasty to assess their symptoms, venous clinical severity, and venous disability. Primary and secondary patency was evaluated with Doppler ultrasound.

**Results**: Seventy-three patients were included in the study. The follow-up of the patients’ clinical symptoms showed significant improvement rates of about 90%, 88.7%, 92.5%, and 100% in claudication, edema, pain, and ulcers-respectively- only 1 month after the procedure. The stent patency rates were 93.2, 91.5, and 92.4 in the 1st, 2nd, 3rd, and 6th postprocedural months, correspondingly. The venous clinical severity score and the venous disability score before the procedure were 14.2 and 2.73, respectively, which were decreased to 5 and 1.1, correspondingly, at 6 months’ follow-up (p value < 0.001).

**Conclusion**: Venoplasty and stenting in our patients with chronic deep venous insufficiency in the lower extremities conferred a significant improvement in clinical symptoms and a high percentage of patency.

## Introduction

Conventional treatment in the chronic deep venous insufficiency of the lower extremities is the use of anticlotting drugs and wearing varices socks. Up to 85% of patients with a previous history of deep vein thrombosis face the post-thrombotic syndrome, manifesting with the onset of pain, swelling, ulcers, and claudication of the lower extremity veins.^1^ About 15% of the patients encounter static lesions, despite antithrombotic treatments.^2^^, ^^3^

The main pathophysiology of venous insufficiency is venous hypertension, which occurs as a result of an obstruction of the deep venous system and the insufficiency of the venous valves. The long-term clinical results of iliofemoral venous insufficiency have been clearly studied.^4^ According to the existing reports, about 44% of patients with previous episodes of chronic venous insufficiency in the lower extremities face symptoms of claudication despite effective treatments.^5^ Thus, the basis for the occurrence of such insufficiency is firstly venous obstruction and secondly, insufficiency of the venous valve.

Chronic iliac vein obstruction in the lower extremities is due to poor compensation by collateral formation, which can lead to severe clinical symptoms.^6^ Before the development of balloon dilatation and stenting-based practices, the treatment of venous obstruction was possible through bypass surgery. The treatment of iliac vein obstruction was traditionally done by femoral bypass or cross-femoral venous bypass (the Palma procedure) or one-way bypass between the distal portion to the obstructed portion of the femoral vein and the iliac vein.^7^ These surgical practices mainly needed long-term anticoagulant therapy and sometimes required an arteriovenous fistula. Although follow-up through venography is necessary in these practices, such follow-up was not done in most clinical studies.^6^

Endovascular treatment is a less invasive modality, and it can be done cutaneously. This method has a high success rate; its complications are very limited, it is accompanied by 1 day’s admission, and the patient returns to normal daily function immediately after the surgery.^8^^-^^11^ In the studies on the efficiency of this method, its patency was reported to be up to 90%.^12^^-^^14^ Also, this treatment method was accompanied by rapid improvement in the patients’ quality of life, and its inefficiency was observed in only 5% of the patients at long-term follow-ups.^15^

Apart from a few studies in the existing literature, information on the results of venoplasty and venous stenting in patients with chronic venous insufficiency in the lower extremities is limited in Iran. This study aimed at investigating the effect of venoplasty and venous stenting in patients with chronic venous insufficiency in the lower extremities in Sina Hospital in Tehran, Iran.

## Methods

This prospective case-series study recruited patients candidated for venoplasty due to chronic venous insufficiency at Sina Hospital, affiliated to Tehran University of Medical Sciences. The study population also had edema, hyperpigmentation, lipodermatosclerosis, and/or venous ulcers. The inclusion criteria comprised significant venous stenosis or obstruction in the proximal veins of the lower extremities on venography, absence of renal failure with creatinine >1.5 mg/dL, and willingness to participate in the study. The exclusion criterion was failure to pass the guide wire from narrowings or blockages in the veins. A study by Neglén et al.^6^ reported the rate of ulcer healing to be 58%. Considering an α of 0.05 and power of 0.8, we estimated the sample size to be 85. Convenient sampling was used, and all patients who met the inclusion criteria were included in the study. The sampling period was from April 2013 to March 2014.

At the beginning of the study, the patients’ primary information-encompassing the demographic characteristics, clinical history, family history, previous history of invasive or noninvasive treatments, type and severity of clinical manifestations, and time elapsed before the procedure-was recorded in a checklist. Additionally, the venous clinical severity score (VCSS)^16^ and the venous disability score (VDS)^17^ in the patients before and after the procedure were recorded. 

The patients who met the inclusion criteria were candidates for venography. Venoplasty and venous stenting was done if stenosis or obstruction was observed in the lower extremity deep venous system or iliac on venography. Next, the patients were evaluated in terms of improvement in their clinical signs and symptoms, VCSS, VDS, and primary and secondary patency rates with Doppler sonography 1, 3, and 6 months postprocedurally. The ethical principles of the Declaration of Helsinki were observed in the current study. Written informed consent for participation in the study was obtained from all the patients. No disturbance occurred in the study population’s diagnostic and therapeutic course, and nor were there any additional costs imposed on the patients. All the participants were entitled to leave the study whenever they deemed fit to do so. 

For the statistical analyses, the statistical software SPSS, version 11, for Windows (SPSS Inc., Chicago, IL) was used. The quantitative variables were compared between the groups using the *t-*test and the repeated measure ANOVA. The chi-square test was employed to compare the qualitative variables. In the statistical analyses, the significance level was considered as a p value < 0.05. 

## Results

Eighty-five interventions were done, and 73 cases were included in the final analysis considering the exclusion criteria (12 cases of failure in the passage of the guide wire). Seventeen (3.2%) patients were female. The mean age of the patients was 42.0 ± 11.5 years (19 to 83 y). There was 1 case of late death, which was irrelevant to stenting and was due to sepsis and respiratory cardiac arrest. Out of 5 cases of early stent obstruction, intervention was done in 3 cases, which was not successful. Two other patients refused to undergo re-intervention.

**Figure 1:**
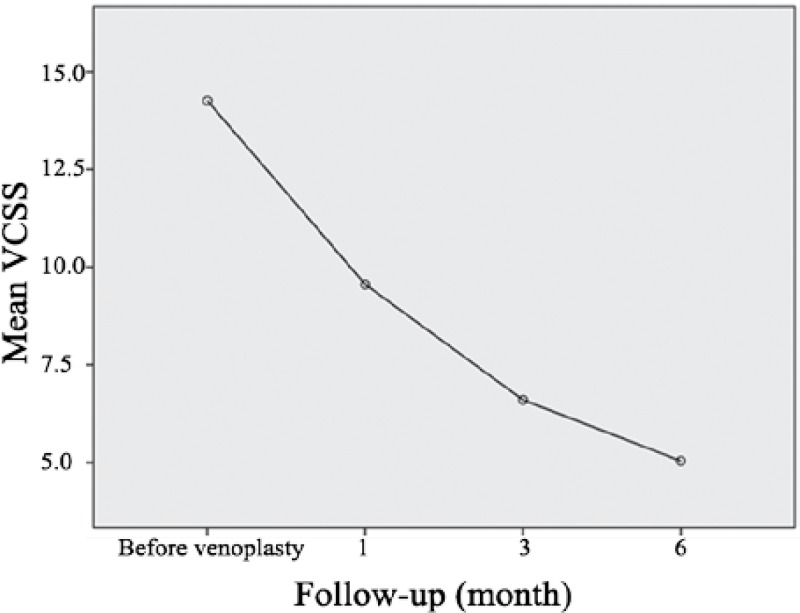
Mean venous clinical severity score (VCSS) before and after venoplasty.

**Figure 2:**
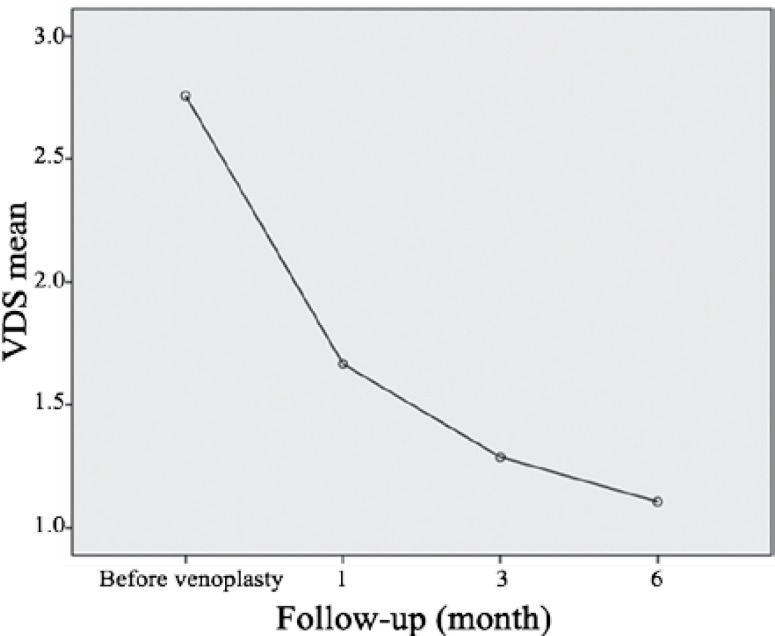
Mean venous disability score (VDS) before and after venoplasty.

The patients were assessed in terms of clinical examination, history of disease, and drug consumption, the results of which are depicted in Table 1. The type of the involved vein, type of the lesion under venoplasty, and type of the stent used in the patients are given in Table 2. Overall, 97 stents at 3 different types and sizes were used for the patients. The frequency of reduced clinical signs and symptoms of the patients in the follow-up is illustrated in Table 3. The mean VCSS and VDS before and after venoplasty and the status of the stents used with respect to patency or in-stent stenosis were assessed so as to investigate venoplasty efficiency; the results are presented in Table 4 and Table 5. The mean VCSS and VDS before and after venoplasty are demonstrated in Figure 1 and Figure 2.

Early complications after intervention were observed in 6 cases: 5 cases of early thromboses, 1 case of hematoma, and 1 case of stent migration. Late complications were observed in 12 cases: 3 cases of stent obstruction, 2 cases of deep vein thrombosis, 2 cases of stomach bleeding, 1 case of varicose, 1 case of epistaxis, 1 case of melena, 1 case of pain, and 1 case of cellulite.

The drugs used after surgery consisted of Clexane^®^, warfarin, and Plavix^®^.

The mean duration of the consumption was 9.2 ± 2.7 days for Clexane^®^, 6.3 ± 4.1 months for warfarin, and 2.4 ± 2.3 months for Plavix^®^.

**Figure 3:**
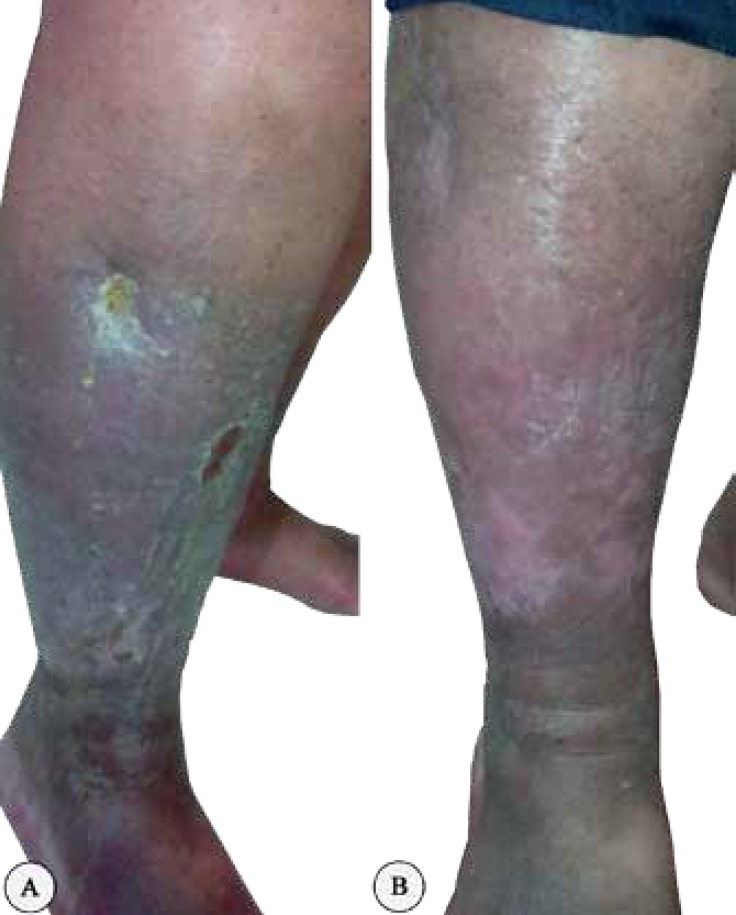
Clinical picture at admission of a 65-year-old male patient with chronic venous insufficiency and multiple ulcers of the right lower leg before (A) and after venoplasty (B).

**Figure 4:**
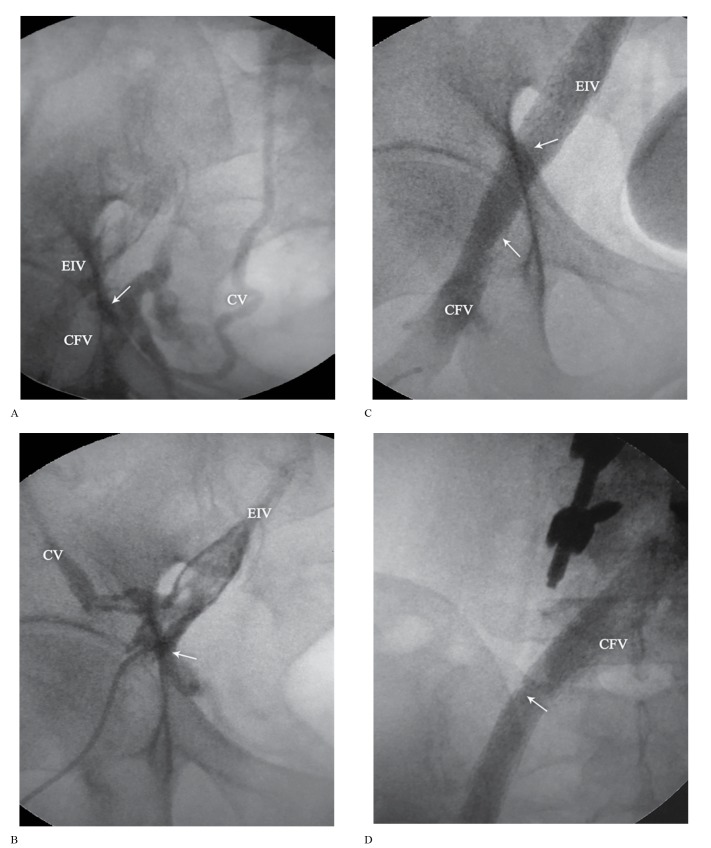
Posteroanterior view of the venography of the venous before (A and B) and after (C and D) venoplasty of the 65-year-old male patient presented in Figure 3.

Figure 3 and Figure 4 demonstrate a sample of the venoplasty results in the patient with venographic evidence.

**Table 1:** Patient Characteristics

	Number	Percentage
Clinical signs and symptoms of affected limbs before venoplasty		
Edema	71	97.3%
Venous claudication	70	95.9%
Localized pain	67	91.8%
Skin color changes	54	74.0%
Varicose vein	42	57.5%
Ulcer	36	49.3%
Lower limb under the procedure		
Left	43	58.9%
Right	30	41.1%
History of disease		
Deep vein thrombosis	43	58.9%
Extremity trauma	19	26.0%
Previous surgery in the affected limb	16	21.9%
Coagulopathies	5	6.8%
Diabetes	2	2.7%
History of drug consumption		
Warfarin	38	52.1%
Clexane^®^	3	4.1%
Triamterene	1	1.4%
Insulin	1	1.4%
Opium	1	1.4%
Smoking	22	31.1%

**Table 2:** Kind of the involved vein, type of the lesion under venoplasty, and the type of the stent used in the patients

	Number	Percentage
Vein of the lower limb under procedure		
Left common iliac	42	57.5%
Right common iliac	25	34.2%
Femoral and common iliac	6	8.3%
Type of the lesion under venoplasty		
Occlusion	48	65.8%
Stenosis	25	34.2%
Type of the stent used (n=97)		
OptiMed (Venous)	56	57.7%
Cordis (S.M.A.R.T.^®^)	29	29.9%
Wall Stent	12	12.4%

**Table 3:** Frequency of reduced clinical signs and symptoms of the patients at follow-up

	One Month’s Follow-Up	Three Months’ Follow-Up	Six Months’ Follow-Up
Frequency of reduced clinical signs and symptoms			
Edema	63 / 71 (88.7%)	58 / 71 (81.7%)	57 / 66 (86.3%)
Venous claudication	63 / 70 (90.0%)	57 / 68 (83.8%)	58 / 64 (90.6%)
Localized pain	62 / 67 (92.5%)	55 / 65 (84.6%)	56 / 62 (90.3%)
Varicose vein	32 / 42 (76.1%)	30 / 41 (73.1%)	35 / 38 (92.1%)
Ulcers	36 / 36 (100%)	34 / 35 (97.1%)	33 / 33 (100%)

**Table 4:** Venous clinical severity score (VCSS) and venous disability score (VDS) before and after venoplasty*

	Before Venoplasty	One Month’s Follow-Up	Three Months’ Follow-Up	Six Months’ Follow-Up	P value
VCSS	14.2±5.7	9.5±4.7	6.7±3.8	5.0±3.7	< 0.001
VDS	2.7±0.5	1.7±0.6	1.3±0.7	1.1±0.6	< 0.001

* Data are presented as mean±SD

**Table 5:** Status of the stents at follow-up*

Stent Status	One Month’s Follow-Up	Three Months’ Follow-Up	Six Months’ Follow-Up
Patency	68 (93.2)	65 (91.5)	61 (92.4)
In-stent stenosis	5 (6.8)	6 (8.5)	5 (7.6)
Total	71 (100)	71 (100)	66 (100)

* Data are presented as n (%)

A) Arrow shows total occlusion of the external iliac vein (EIV) and stenosis of the common femoral vein (CFV) before venoplasty; blood flow is drained through the collateral vein (CV).

B) Arrow shows total occlusion of the external iliac vein and formation of collateral vein.

C) Arrows show resolution of the occlusion of the external iliac vein and stenosis of the common femoral vein after venoplasty and stent placement.

D) Arrow shows resolution of the occlusion of the common iliac vein after venoplasty and stent placement.

## Discussion

Venous outflow obstruction plays a significant role in the emergence of clinical symptoms of chronic venous disease. In the past decade, percutaneous endovenous stenting has been considered an appropriate treatment protocol for venous outflow obstruction. The stenting of venous outflow obstruction in the lower extremities may lead to a high rate of patency and a low rate of restenosis.^12^

Since the long-term outcome of stenting depends on various factors such as etiologic factors and patients’ clinical condition, in the present study, the clinical symptoms of patients with chronic venous insufficiency in the lower extremities were examined before and after venoplasty in order to determine the rate of improvement in these symptoms. The highest rate of reduction in clinical symptoms was related to ulcers at 1 month’s follow-up (100%) and the lowest reduction rate was observed in varicose veins (76.1%). The 1-month follow-up of the patients demonstrated a reduction in the clinical signs and symptoms inasmuch as claudication, edema, and pain showed specific reductions of about 90%, 88.7%, and 92.5% - respectively - only 1 month after the procedure. The improvement trend and the reduction in the clinical signs and symptoms in the patients at 3 and 6 months’ follow-ups continued, so that total improvement in the clinical symptoms was observed in some cases. 

The other important variable considered in the current study was the patency rate in the patients. The patency rates in the 1st, 3rd, and 6th postprocedural months were 93.2%, 91.5%, and 92.4%, respectively. Similar studies have examined patency in the follow-up of patients. Raju et al.^18^ performed balloon dilatation to eliminate chronic iliac vein stenosis in their patients and reported that during a 24-month follow-up, the primary and secondary patency rates were 71% and 90%, correspondingly. In addition, the percentage of limbs without edema was increased to 47% from 12%, and the pain score was reduced to 1 from 4 after the treatment. Finally, the authors reported that venous ulcer healing reached 62% during their 24-month follow-up period. Neglén et al.^19^ conducted 99 stenting procedures of iliofemoral veins on 99 limbs and reported that at 5 years’ follow-up, 27% of their study population had deep axial reflux and there was no death. The investigators also reported that the cumulative, primary, and secondary patency rates were 83%, 97%, and 97% during a 4-year period. Moreover, edema and limb pain were significantly reduced and ulcer healing was observed in 68% of the patients. Hartung et al.^20^ studied patients with chronic venous insufficiency under endovascular treatment in a period of between 3 and 10 years and reported accumulative, primary, and secondary patency rates of 83%, 89%, and 93%, respectively. In their study, the occurrence of deep vein thrombosis was the main effective factor in the prediction of patency. In another study, Hartung et al.^21^ studied patients under treatment with chronic inefficiency or iliocaval vein occlusion and reported accumulative, primary, and secondary patency rates of 73%, 88%, and 90%-correspondingly-during a 36-month period. In another investigation, Hartung et al.^22^ reported accumulative, primary, and secondary patency rates in endovascular treatment with or without stents during a 36-month period of 79%, 86%, and 86%, respectively. The authors also reported a success rate of 95.5% and a stent migration rate of 4.5%.

In the current study, the VCSS and VDS showed significant reductions at follow-up times compared to the values before intervention. Finally at 6 months’ follow-up, the VCSS of the patients reached 5 from 14.2 and the VDS reached 1.1 from 2.7. Similar results were obtained in other studies. For example, Hartung et al.^22^ found that the VCSS reached 3 from 10.5 and the VDS reached 1 from 2.5. Also, in another study by Hartung et al.,^21^ it was found that the VCSS reached 2 from 8.5 and the VDS reached 0 from 2.

In terms of complications in the present study, the highest rates belonged to thrombosis and stent obstruction. In the studies by Hartung et al.,^20^^–^^22^ delayed death, venous thrombosis, and stenosis were reported to be the most frequent complications.

## Conclusion

Considering the findings obtained in the current study, it can be concluded that venoplasty and venous stenting in patients with chronic venous insufficiency in the lower extremities leads to considerable improvement in the clinical signs and symptoms and confers high rates of patency.

We would recommend that randomized clinical trials be conducted in different medical centers to further assess the outcome of venous venoplasty and the effects of the underlying variables on the treatment outcome. 

## Notes:

***This paper should be cited as:*** Moeini M, Zafarghandi MR, Shahbandari M, Sayarifard A, Taghavi M, Salimi J, Farshidmehr P, Hasani M, Tobaei MR. Venoplasty and Venous Stenting in Patients with Chronic Venous Insufficiency in the Lower Extremities. J Teh Univ Heart Ctr 2016;11(4):174-180.
